# Diversifying selection and functional analysis of interleukin-4 suggests antagonism-driven evolution at receptor-binding interfaces

**DOI:** 10.1186/1471-2148-10-223

**Published:** 2010-07-22

**Authors:** Madoka Koyanagi, Julie A Kerns, Linda Chung, Yan Zhang, Scott Brown, Tudor Moldoveanu, Harmit S Malik, Mark Bix

**Affiliations:** 1 Department of Immunology, St. Jude Children's Research Hospital, 262 Danny Thomas Place, Memphis, TN 38105 USA; 2 Division of Basic Sciences, Fred Hutchinson Cancer Research Center, 1100 Fairview Ave N, PO Box 19024, WA 98109 USA; 3 HHMI, Fred Hutchinson Cancer Research Center, 1100 Fairview Ave N, PO Box 19024, WA 98109 USA; 4 Division of Viral Immunology, Center for Aids Research, Kumamoto University, 2-2-1, Honjo, Kumamoto 860-0811 Japan; 5 Institute for Systems Biology, 1441 North 34th Street, Seattle, WA 98103 USA; 6 Trubion Pharmaceuticals, 2401 4th Ave, Seattle, WA 98121 USA

## Abstract

**Background:**

Interleukin-4 (IL4) is a secreted immunoregulatory cytokine critically involved in host protection from parasitic helminths [1]. Reasoning that helminths may have evolved mechanisms to antagonize IL4 to maximize their dispersal, we explored mammalian IL4 evolution.

**Results:**

This analysis revealed evidence of diversifying selection at 15 residues, clustered in epitopes responsible for IL4 binding to its Type I and Type II receptors. Such a striking signature of selective pressure suggested either recurrent episodes of pathogen antagonism or ligand/receptor co-evolution. To test the latter possibility, we performed detailed functional analysis of IL4 allotypes expressed by *Mus musculus musculus *and *Mus musculus castaneus*, which happen to differ at 5 residues (including three at positively selected sites) in and adjacent to the site 1 epitope that binds the IL4Rα subunit shared by the Type I and Type II IL4 receptors. We show that this intra-species variation affects the ability of IL4 neither to bind IL4 receptor alpha (IL4Rα) nor to signal biological responses through its Type I receptor.

**Conclusions:**

Our results -- reminiscent of clustered positively selected sites revealing functionally important residues at host-virus interaction interfaces -- are consistent with IL4 having evolved to avoid recurrent pathogen antagonism, while maintaining the capacity to bind and signal through its cognate receptor. This work exposes what may be a general feature of evolutionary conflicts fought by pathogen antagonists at host protein-protein interaction interfaces involved in immune signaling: the emergence of receptor-binding ligand epitopes capable of buffering amino acid variation.

## Background

Viruses and bacteria have evolved survival strategies based on antagonism of host immunoregulatory molecules [2]. Cytokine signaling pathways are prime targets, often subverted by horizontal acquisition of genes encoding cytokines or their receptors that are then selectively modified and marshaled [3,4]. With genomes orders of magnitude larger than viruses and bacteria, helminths have the capacity to maintain multiple and complex immune antagonizing strategies to facilitate their intricate life cycles within obligate mammalian hosts. Indeed, recent work has shown that filarial nematodes express a homolog of transforming growth factor beta (TGFβ) that can bind to host receptors [5]. Nevertheless, compared to bacteria and viruses, our knowledge of immune modulatory mechanisms employed by parasitic helminths is in its infancy.

Interleukin-4 (IL4) is a 17 kDa monomeric glycoprotein of the Type I hematopoietin superfamily secreted by T helper 2 (Th2) cells, NK T cells, mast cells and basophils [6-9]. Its pleiotropic functions are still being enumerated and include acting as a Th2 cell developmental determinant, a T/B cell growth factor, an IgE/IgG1 class-switch inducer and a muscle cell contraction inducer. Each of these functions of IL4 plays a role in mobilizing and coordinating anti-helminth immune responses [10]. *In vivo *administration of antibodies or recombinant cytokines that raise IL4 levels diminish helminth infection; conversely, lowering IL4 levels increases helminth infection [11,12]. Mice genetically deficient in IL4 or the IL4 receptor display impaired capacity to control experimental worm infection [1]. IL4 transduces signals to the cell interior via two distinct heterodimeric receptors that share a 140-kDa membrane-spanning IL4Rα subunit [6,13]. IL4Rα is paired in the Type I receptor with the 42-kDa common gamma subunit (γc) and in the Type II receptor with the 65-kDa interleukin-13 receptor alpha 1 subunit (IL13Rα1). The Type II receptor binds not only IL4 but also the paralogous cytokine IL13. Through alternative splicing, both IL4Rα and IL13Rα1 can be secreted as soluble molecules that can modulate immune responses by altering local concentrations of free IL4 and IL13 [14]. This complex receptor system mediates the pleiotropic functions of IL4 through regulated and non-uniform expression on a wide variety of hematopoietic and other cell types.

As a key host molecule triggered by and required for the control of parasitic worms [10], IL4 may constitute a battleground upon which helminths are locked in evolutionary conflict with their mammalian hosts. Testifying to the crucial adaptive nexus occupied by IL4, evolutionary diversifying selection has been detected acting on the human *IL4 *promoter, leading to the fixation of promoter sequence variants that differentially bind the transcription factor NFAT and consequently have distinct thresholds for transcriptional triggering [15]. High levels of IL4 expressed by individuals inheriting the sensitive form of the promoter might provide a mechanism to overwhelm a parasite-derived IL4-antagonist molecule.

Previous studies have remarked on the rapid evolution of IL4 and IL4Rα [16-18]. To explore whether pathogen antagonism could provide an explanation, we analyzed IL4 sequences from multiple mammalian lineages and found a strong signature of diversifying selection, clustered at interfaces that interact not only with IL4Rα but also with IL13α1. This striking evolutionary signature is consistent with pathogen antagonism of IL4 but formally is also consistent with rapid co-evolution between IL4 and its receptor. We tested the latter possibility by exploiting the fact that in mice, natural intra-specific IL4 variation clusters at the site 1 epitope for IL4Rα binding. In sensitive cellular and biochemical studies of two murine IL4 allotypes, we found that site 1 epitope variation is functionally neutral with respect to IL4Rα binding affinity and kinetics as well as signalling IL4-dependent cellular responses. Given the functional neutrality of intra-specific variation at 3 residues evolving under diversifying selection, we speculate that the capacity to buffer amino acid variation at the IL4Rα site 1 epitope is an adaptation to recurrent pathogen antagonism, seen previously in other host-host interaction surfaces subject to pathogen mimicry [4,19].

## Results

### Evolution of IL4 in mammals

**Figure 1:**
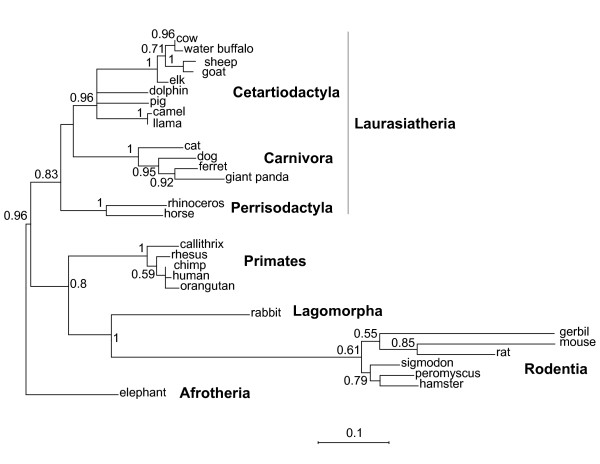
**Phylogenetic sequence analysis of IL4 from mammalian lineages**. Shown is a neighbor-joining tree based on a nucleotide alignment (Additional File 3) of IL4 from the 28 eutherian mammals. The presented PhyML maximum likelihood phylogeny (using the HKY85 model) is rooted using elephant IL4. Numbers at branching nodes indicate the bootstrap support for that branch (1 = 100%). The orders of eutherian mammals are also indicated in bold with the Afrotheria (elephant) branch shown as the basally separating branch among the Eutheria [22].

**Figure 2:**
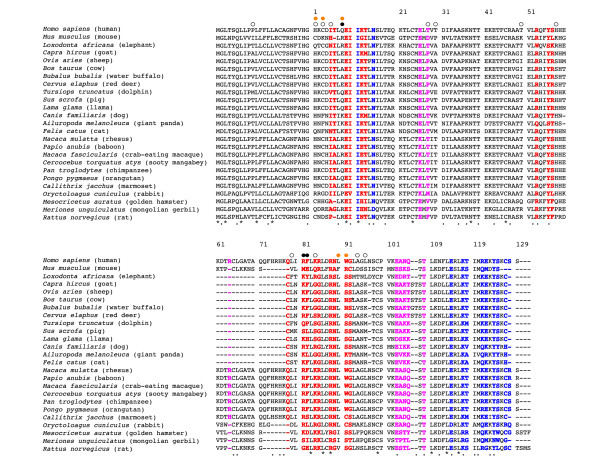
**Alignment of IL4 protein sequences from mammalian lineages**. Site 1, site 2 and site 3 epitope residues important for interaction with IL4Rα, γc and IL13Rα1 are colored red, blue and magenta, respectively. *M. musculus *and *M. castaneus *polymorphic residues (orange circles) and positively selected residues (open circles, black circles for sites with [P] > 0.95) are indicated at the top of the alignment. Conserved residues are indicated at the bottom of the alignment, as either identical (asterisks) or similar (dots).

Diversifying or positive selection is typically seen in molecular arms races, especially at direct interfaces between host and pathogen proteins [4,20]. We investigated whether sequence variation of IL4 could have arisen by recurrent diversifying selection (perhaps shaped by pathogen antagonism) during mammalian evolution. We used data from whole genome sequencing projects to compare the *IL4 *coding sequence from 28 eutherian mammalian lineages. The 'Model Selection tool' at the online HyphY server (see methods) identified the HKY85 model as the most appropriate using the AIC criterion (p <0.07). A maximum-likelihood phylogeny (Figure 1) was constructed using the PhyML method using the IL4 coding nucleotide alignment; this phylogeny was largely congruent with accepted mammalian phylogeny [21,22]. An almost identical topology was obtained using distance based neighbor-joining methods, except for minor variation in the Rodentia. Figure 2 shows the amino acid alignment of representative IL4 proteins from 25 mammals, including 7 primates. Using the free-ratio model in the PAML suite of programs [23], which allows an independent assignment of dN/dS ratios to each evolutionary branch, we found that most branches of the phylogeny show dN/dS <1 with the overall dN/dS ratio for the entire tree equaling 0.66 (2.33/3.68). The accelerated evolution of the rodent lineage [24] is evident in the long branch leading to the four rodents but this is not due to an overall increase in dN/dS in rodents. Indeed, the free-ratio model (in which all branches can have different dN/dS ratios) was not a statistically superior fit to the one-ratio model (2lnλ = 60.73, degrees of freedom = 53, p > 0.2)

When we compared the likelihood of *IL4 *evolution under codon models that prohibit (NSsites model M7) or permit diversifying selection (M8), we found that models permitting diversifying selection fit *IL4 *evolution significantly better than those that disallow it (Table 1). In the M7-M8 comparison, we found that 15% of residues have evolved with an average dN/dS ratio of 1.8 indicating that IL4 has evolved under recurrent diversifying selection in mammals (Table1, p < 10^-4^) consistent with previous reports [16-18,25]. We found robust evidence of positive selection in an analysis limited to rabbits and rodents alone, limited to Laurasiatheria alone, and to all eutherian mammals excluding either rodents and rabbits, or excluding primates. However, we do not find robust evidence for diversifying selection when the analysis is limited to 16 primates including 2 prosimians (2lnλ = 0.73, *p *> 0.5, Table 1). This suggests that the selective pressure that recurrently drove positive selection of eutherian IL4 may be relaxed in the primate lineage. A recent primate evolution study of genes involved in HIV pathogenesis also found evidence for IL4 having experienced diversifying selection [16]. The discrepancy with our IL4 analysis [that failed to detect significant evidence of diversifying selection when restricted to primates alone (Table 1)] may be due to different species analyzed in each study. It must also be noted that the signature for selection in the previous study was modest [16] with only 1 site identified as having evolved under positive selection.

**Table 1:** Summary Statistics of PAML analyses

NSsites Model comparison	2 lnλ (df)	p-value	%codons dN/dS >1	average dN/dS
M7 vs M8, eutherian mammals- 28 taxa (including elephant, carnivores, rabbit, rodents, primates)	**20.84 (2)**	**<10**^**-4**^	15.2	1.87

M7 vs M8, eutherian mammals excluding Lagomorphs & Rodentia (21 mammals)	**20.61 (2)**	**<10**^**-4**^	16.7	2.14

M7 vs M8, Lagomorphs & Rodentia alone (7 taxa)*	**19.56 (2)**	**<10**^**-4**^	5.6	6.34

M7 vs M8, primates alone (16 taxa incl. 2 prosimians)	0.737 (2)	>0.5	na	na

M7 vs M8, eutherian mammals excluding primates (23 taxa)	17.28 (2)	**<0.0002**	18.5	1.75

M7 vs M8, Laurasiatheria alone (15 taxa)	**13.15 (2)**	**<0.002**	16.3	2.02

All analyses were done using the Fcodon model. Results are qualitatively identical using the F3 × 4 model of codon substitution.

lnλ = log likelihood difference between the two models

df = degrees of freedom

*Multiple topologies for rodent phylogenies were tested due to poor phylogenetic resolution in this lineage - the conclusion of positive selection is not dependent on the use of a particular topology; the values reported are those found when used with a phylogeny consistent with the consensus view of rodent evolution.

Our analysis identifies 15 codon positions in which there is significant evidence of diversifying selection (posterior probability >0.5 under a Naïve Empirical Bayes model) (open circles in Figure 2) of which three sites meet the more stringent criteria of posterior probability >0.95 (closed circles in Figure 2). These sites are also identified with high confidence under a Bayes Empirical Bayes model [26]. Despite the long history of IL4 evolution sampled here, several positions are either invariant or very similar in eutherian mammals (highlighted with asterisks and dots, respectively).

IL4 folds into a 4-helix bundle structure similar to that of GMCSF, IL2 and IL5 [6]. The structural epitope for IL4Rα binding (called site 1) is large (1560 Å), comprising a mosaic of 18 residues contributed by alpha helices A, B and C and mediates high affinity binding (Kd ~ 1 nM) [6,13]. Directly adjacent to site 1 is a 1070 Å site 2 epitope for γc binding comprising 10 residues contributed by alpha helices A and D [6,13]. The IL13Rα1 subunit found in the Type II IL4 receptor expressed on non-hematopoietic cells binds to a distinct 740 Å site 3 epitope comprising 8 residues contributed by loops connecting helices A-B and C-D on the IL4 face opposite site 1 [13]. Both the γc and IL13Rα1 subunits exhibit low affinity for the binary IL4/IL4Rα complex (Kd ~ 500 nM) and even lower (mM) affinity for soluble IL4. Accordingly, a two-step model has been proposed for IL4 binding to its Type I and II receptors [6,13]. In an initial 3D reaction, IL4Rα binds with high affinity to soluble IL4 via the site 1 epitope. In a second 2D reaction restricted to the plane of the cell membrane, the binary IL4/IL4Rα complex binds γc in the Type I receptor or IL13Rα1 in the Type II receptor via site 2 or site 3, respectively [6,13].

**Figure 3:**
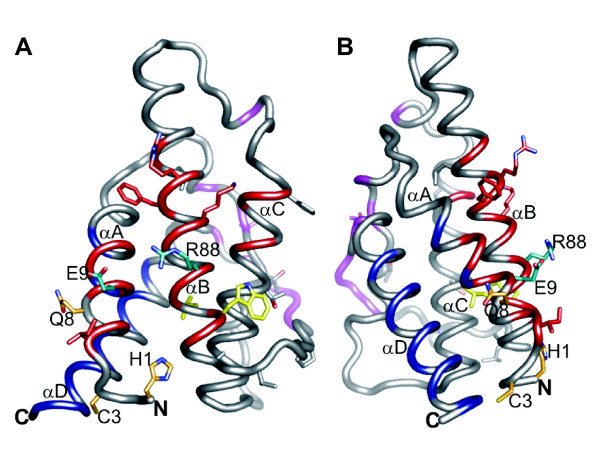
**Analysis of murine IL4 variation**. Cartoon representations depicting human IL4 structure (PDB ID: 1H1K) were rotated (90°) to feature (A) the site 1 epitope (red) and (B) the site 2 epitope (blue). Side chains are shown for residues that are non-synonymously substituted between *M. musculus *and *M. castaneus *(yellow and orange), positively selected (H1, C3 and Q8; orange), and that comprise the mixed-charge pair responsible for the majority of the IL4Rα binding energy (E9 and R88; cyan). Site 3 epitope residues are shown in magenta. Alpha helices A, B, C and D are labeled. (All structure representation were created in PyMol, http://www.pymol.org)

Strikingly, positively selected residues cluster in two groups (Figures 2 and 3) that overlap site 1 (highlighted in red) and site 3 (highlighted in magenta) epitopes. That clustering occurs at the IL4 ligand:receptor interface could suggest that sequence variation was driven by selective pressure to modulate signaling intensity. Alternatively, sequence variation may be driven by pathogen antagonism to directly interfere with IL4: IL4Rα interactions; in the latter case, sequence variation would be expected to be functionally neutral for ligand:receptor interactions so as to avoid disadvantageous perturbations to normal IL4 signaling. To distinguish between these possibilities, we decided to test the functional consequences of sequence variation for IL4: IL4Rα interactions, focusing on recently diverged murine homologs of IL4 that had alterations specifically at their receptor interacting residues.

### Clustered intra-specific variation at the IL4 site 1 epitope is functionally neutral for IL4Rα binding and signalling

**Figure 4:**
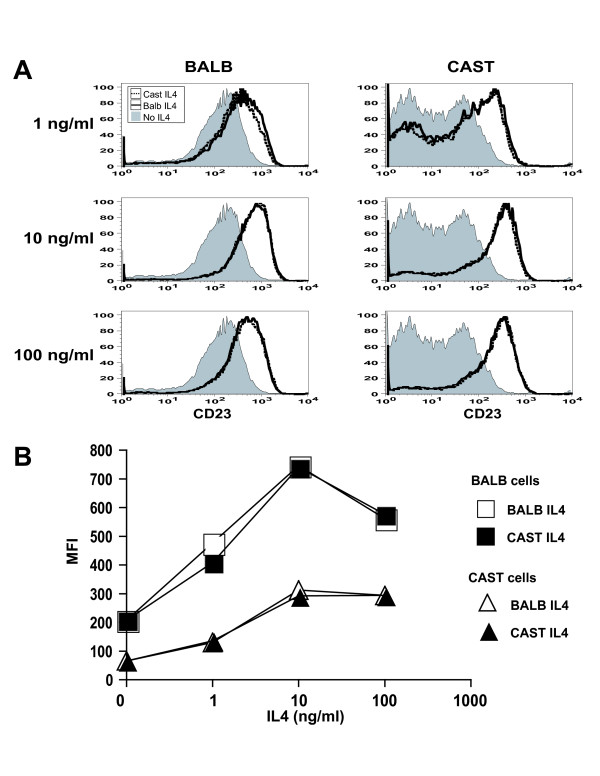
**CD23 expression induced by BALB/c and CAST/Ei IL4**. (A) Shown are representative FACS plots depicting CD23 expression level on B cells isolated from BALB/c and CAST/Ei mice and stimulated for 16 hr with graded concentrations of BALB/c and CAST/Ei IL4. (B) Shown is quantification of mean fluorescence intensity (MFI) versus dose of the data in (A). Data are representative of 3 independent experiments with similar results.

We compared the murine sub-species *M. musculus *(strain BALB/c) and *M. castaneus *(strain CAST/Ei) and discovered 5 amino acid sequence changes between them (Additional File 1). To infer the spatial distribution of the 5 polymorphic residues we aligned human and mouse IL4 sequences and mapped orthologous residues onto the human IL4 protein structure (PDB ID: 1HIK) (Figure 3). Strikingly, the 5 polymorphic residues (Figure 4: Mouse D2, K3, R8, F90, R91 and human K2, C3, Q8, N89, L90; Table 2, numbering from NP_067258) mapped either in or adjacent to the IL4Rα-binding site 1 epitope.

**Table 2:** Biacore analysis of BALB and CAST IL4 binding to IL4Rα

	**k**_**a**_**(1/Ms)**	**k**_**d**_**(1/s)**	**K**_**A**_**(1/M)**	**K**_**D**_**(M)**
BALB IL4	2.49E+06	5.18E-04	4.79E+09	2.09E-10

CAST IL4	3.16E+06	7.13E-04	4.44E+09	2.26E-10

Data are the average of two independent experiments performed with two independently coupled chips.

k_a _is the association rate constant (M^-1^s^-1^);

k_d _- is the dissociation rate constant (s^-1^);

K_A _- is the equilibrium binding (affinity) constant (k_a_/k_d_) (1/M);

K_D _- is the equilibrium dissociation constant (k_d_/k_a_) (M)

To test whether naturally arising variation in site 1 residues is functionally relevant with respect to IL4Rα interactions, we expressed and purified recombinant BALB/c and CAST/Ei IL4 for biophysical and functional studies. To determine the affinity of IL4Rα for BALB/c and CAST/Ei IL4, we measured surface plasmon resonance (SPR) using a Biacore apparatus. Purified *M. musculus *IL4Rα extracellular domain [27] was immobilized on an SPR chip and graded doses of recombinant IL4 were passed in the mobile phase over the chip. Surprisingly, BALB/c and CAST/Ei IL4 bound IL4Rα with similar affinity (Kd ~200 pM,) and kinetic on and off rates (Table 2 and Additional File 2), in good agreement with previously published measurements [6,13].

**Figure 5:**
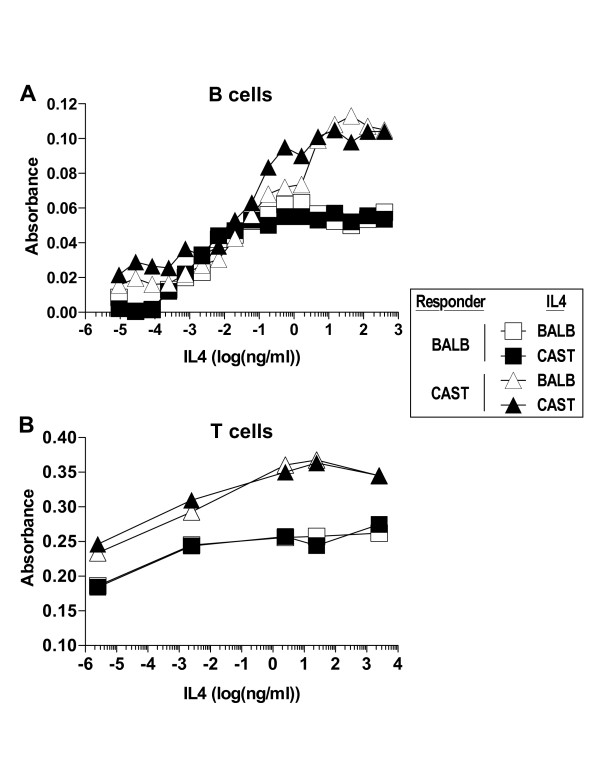
**Proliferative response of B and T cells to BALB/c and CAST/Ei IL4**. (A) Shown are plots depicting the proliferative response of B cells from BALB/c (left panel) and CAST/Ei (right panel) stimulated with 50 ng/ml of LPS in the presence of graded concentrations of BALB/c (open) or CAST/Ei (closed) IL4. (B) Shown is a plot depicting the proliferative response of T cells from BALB/c (square) and CAST/Ei (triangle) cultured in the presence of graded concentrations of BALB/c (open symbols) or CAST/Ei (closed symbols) IL4. Proliferation was measured as the rate of AlamarBlue reduction. Data are the mean of duplicate measurements and are representative of 3 independent experiments with similar results.

Next, we sought to determine whether, despite sharing similar binding affinity for IL4Rα, BALB/c and CAST/Ei IL4 might differ in their ability to signal IL4-dependent biological responses. Mouse B and T cells fail to express IL13α1 and therefore express only the Type I IL4 receptor [28], ensuring their readouts will be sensitive to IL4Rα interactions. First, we compared the capacity of BALB/c and CAST/Ei IL4 to signal B cells to upregulate cell surface expression of CD23, a well-established IL4-dependent response [29-31]. Over a 1000 fold dynamic range we detected no difference in the ability of BALB/c and CAST/Ei IL4 to signal CD23 upregulation, regardless whether the responding cells were of BALB/c or CAST/Ei origin (Figure 5). As IL4 is also a B and T cell growth factor [32], we tested its relative capacity to induce cellular proliferation. As with upregulation of cell surface molecules, BALB/c and CAST/Ei IL4 elicited identical proliferative responses from both B and T cells (Figure 5A and 5B), again regardless whether the responding cells were of BALB/c or CAST/Ei origin. Taken together, these results indicate that naturally arising genetic variation in IL4 residues mapping to the site 1 epitope is functionally neutral with regard to IL4Rα binding and biological signalling.

**Figure 6:**
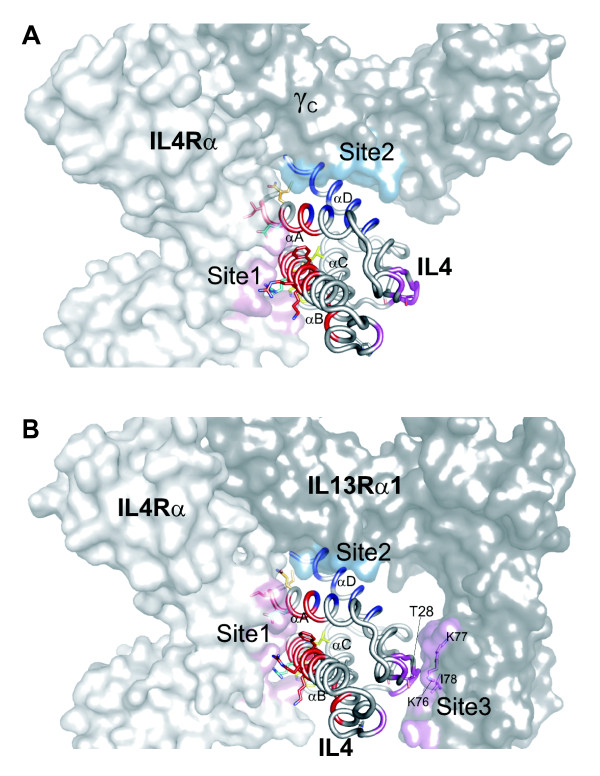
**Mixed cartoon-surface representations of receptor/ligand complexes of (A) Type I IL4 ternary complex (PDB ID:**3BPL) **comprising IL4Rα:IL4:γ**_**C**_** and (B) Type II IL4 ternary complex (PDB ID:**3BPN) **comprising IL4Rα:IL4:IL13Rα1**. Receptor residues making van der Waals contact (<4 Å) with residues in IL4 site 1, site 2, and site 3 epitopes are highlighted in pink, blue and violet, respectively. Labeled is positively selected residue T28 in IL4 site 3 and IL13Rα1 site 3-binding residues (K77, K76 and I78). Coloring scheme for IL4 was retained from Figure 3.

Our finding that intra-specific changes in IL4 were functionally neutral for IL4 receptor binding suggests a high degree of redundancy and therefore relaxed constraint at the ligand:receptor interface. This is at apparent odds with our evidence for diversifying selection acting at 3 of 5 site 1 epitope residues polymorphic between *M. musculus *and *M. castaneus *(Figures 2 and 6). Similar failures to detect functional ramifications of variation at sites identified as subject to recurrent diversifying selection were reported recently in studies of environmental adaptation -- detection of light wavelengths by rhodopsin [33] and odors by an odorant receptor [34]. However, selective episodes driving environmental adaptation are expected to be of low frequency and intensity, leaving variants associated with very subtle functional ramifications. By contrast, selective episodes driven by classical Red-Queen host:pathogen conflicts are expected to be of high frequency and intensity, leaving variants associated with robust functional ramifications [35]. Importantly, this rapid evolution would be expected at host:pathogen not host:host interfaces, entirely consistent with our functional data. Given that PAML methods [23] and especially clustering of residues subject to diversifying selection have proven diagnostic not only of antagonism but also of host specificity determinants [19,20,36,37], and given the important role played by IL4 in host protection from helminths, it is likely that the evolutionary signatures we have discovered point to a previously hidden mode of pathogen antagonism, although the identity of this antagonist(s) currently remains unknown.

## Discussion

Our analyses are consistent with previous studies that used human population genetics and SNP association to suggest that IL4 and IL4Rα have experienced recent episodes of diversifying selection [15,17]. Additional support comes from a recent phylogenetic sequence analysis comparing human, primate and mouse that detected signatures of diversifying selection (high R/S values) for IL4 and other γc-dependent cytokines (IL2, IL7, IL9, IL15 and IL21). Based upon the failure to detect a similar signature of diversifying selection in γc itself, the authors proposed that cytokine selection was driven by mutual competition for γc, a common receptor subunit for each cytokine [25]. Consistent with O'Connell et al [25], we did not detect positively selected codon positions in the IL4 γc-binding site 2 epitope. However, the prediction that flows from the O'Connell hypothesis -- that IL4 variants differing in positively selected residues would bind with differential affinity to IL4Rα -- is not borne out by our functional data.

The earliest support for IL4 having evolved under diversifying selection comes from pair-wise analyses of mouse and rat IL4 and IL4Rα that detected elevated R/S ratios in the receptor-binding region of IL4 and the extracellular (but not cytoplasmic) domain of IL4Rα [18,38]. The authors of those studies concluded diversifying selection of IL4 was driven by co-evolution of the receptor/ligand pair [18,38]. But, co-evolutionary forces tend to impede rather than facilitate evolutionary changes at protein-protein interaction surfaces.

We propose instead that genetic conflict (for example, recurrent adaptation to escape pathogen antagonism) is most consistent with such evolutionary signatures. We have found that IL4 has evolved under strong and recurrent diversifying selection focused at 15 positions (with a NEB criteria). Twelve of these fall in or near the site 1 epitope comprising the IL4Rα binding interface. Two more positively selected residues form a second cluster falling in or near the site 3 IL13Rα1-binding epitope used by the Type II IL4 receptor. From an evolutionary perspective the interaction interface of IL4 with its receptor is an ideal antagonist target as it is likely to be functionally constrained, necessitating escape from antagonism to require coordinated changes in both IL4 and its receptor. It is tempting to speculate that pathogen antagonism has specifically targeted IL4 complexed with the non-hematopoietic Type II IL4 receptor (that lacks γc), as would be the case for gut and lung resident helminths confronting Type II IL4 receptor-dependent expulsatory effector mechanisms triggered by IL4 activation of intestinal and lung epithelia [39,40].

A functional comparison of two intra-specific murine IL4 proteins allowed a direct evaluation of the functional consequences of variation at positively selected codon positions without the confounding changes that may occur due to epistasis over long evolutionary divergences (wherein functional consequences of changes at one position might have been buffered or masked by evolutionary changes in another). Our discovery that genetic variation at three such positively selected residues in murine IL4 is functionally neutral with respect to both IL4Rα binding and signaling suggests that the IL4 site 1 epitope displays a remarkable degree of evolutionary flexibility to accommodate changes in sequence to escape (inferred) antagonism by parasite-encoded proteins while maintaining interaction with its receptor.

Approximately 80% of the IL4Rα binding energy for IL4 comes from interactions with a core of 2 residues (Figure 3 and 6; E9 and R88) [41]. Interestingly, these two residues are not invariant in mammalian evolution, which is what might have been expected if this interface were not subject to antagonism [42] (Figures 1 and 6). Consistent with our hypothesis, alanine scanning mutational analysis has shown that the remaining 16 residues comprising the site 1 epitope and collectively accounting for the remaining binding energy can accommodate significant amino acid changes without destroying IL4Rα binding [6,41,43]. This arrangement may serve to buffer ligand/receptor allelic polymorphism, thereby creating a reservoir of subtly modified interfaces upon which diversifying selection can act to efficiently identify IL4 variants that have lost the ability to interact with parasite-derived IL4-binding molecules. Indeed, we can argue that constant antagonism of this interaction interface may have itself selected for this highly diffuse interaction surface; the more diffuse or flexible the IL4-IL4Rα interaction, the easier it would be to emerge victorious from an evolutionary episode of Darwinian selection against an antagonist. It will be interesting to determine whether, like the site 1 epitope used by the IL4Rα subunit, the site 3 epitope will be able to tolerate amino acid variation without perturbing IL13Rα1 binding.

## Conclusions

We posit that the striking signature of diversifying selection coupled with the absence of a functional consequence on the IL4:IL4Rα interaction provides strong evidence for the existence of a pathogen antagonist that shapes IL4 evolution. A precedent for our hypothesis comes from a case study of Protein Kinase R (PKR) and its highly conserved substrate eukaryotic translation initiation factor 2 alpha (eIF2α). Pathogen mimics of eIF2α can antagonize the eIF2α:PKR interaction. A key to escape this antagonism may be the evolution of a highly flexible PKR epitope responsible for recognition of eIF2α [19,37]. Similarly, we propose that frequent and intense pathogen antagonism may have fixed an interaction strategy based upon the buffered epitope that characterizes the interaction of IL4 with IL4Rα (and, perhaps, also of IL4:IL4Rα with IL13Rα1) [4]. Numerous other immune protein:protein interactions bear striking resemblance to the diffuse flexible IL4 site 1 epitope that binds IL4Rα. It is interesting to speculate that these interfaces also have been borne out of evolutionary conflicts to escape pathogen subversion. Validation of this scenario awaits discovery of a pathogen (helminth)-derived immune antagonist molecule capable of binding and neutralizing IL4. Based on the location of positively selected residues, it would not be surprising if this antagonist were a mimic of either IL4 or IL4Rα [4,19].

## Methods

### Evolutionary comparison of IL4 across mammals and primates

We used TBLASTN and BLASTN searches [44] using either the mouse or human IL4 protein to identify all full-length eutherian mammal IL4 sequences in either nr (non-redundant), wgs (whole genome shotgun), est (expressed sequence tags) or htgs (high throughput genome sequencing) databases. These sequences were translated and the amino acid sequences aligned using CLUSTAL_X [45]. The amino acid alignments were then converted to nucleotide alignments using the PAL2NAL program [46]. Neighbor-joining phylogenies based on the nucleotide alignment were constructed and bootstrapped using the CLUSTAL_X program. Using the 'Model Selection' tool on the online HyPhy server (http://www.datamonkey.org), we first identified the appropriate nucleotide substitution model, in this instance HKY85. Maximum likelihood trees were constructed and bootstrap analyses performed using the PhyML program [47,48] and either HKY85 or GTR models of nucleotide substitution [49]. Both models of nucleotide substitutions gave identical results with PhyML. Trees were visualized using the Dendroscope Program [50]. This nucleotide alignment (that honors codon definitions) was used as an input for tests of diversifying selection. We used both an alignment of 28 mammalian representatives and 16 simian primates alone for our analysis. Both these alignments are presented in Additional Files 3 and 4.

We tested the dataset for evidence of recombination using two programs in the HyPhy suite [51] (http://www.datamonkey.org[52]): GARD and SBP [53]. Neither program found evidence for any recombination in the IL4 dataset (cAIC criterion for SBP). Predictions of individual codons that evolved under diversifying selection were performed by maximum likelihood using NSsites model comparisons in PAML [23]. To identify diversifying selection, we required a significant difference in log likelihoods, by chi-squared testing, between nested PAML models M7 and M8. Model M7 allows codons to have dN/dS values according to a beta distribution (two parameters). Model M8 is the same as M7 except that it adds a discrete category of dN/dS with dN/dS >1. Significance of diversifying selection was tested by comparing twice the difference in log likelihoods (2lnλ) between M7 and M8 with two degrees of freedom. Codons predicted to be under diversifying selection were identified by Naïve-Empirical-Bayes (NEB) and Bayes-Empirical-Bayes (BEB) analysis. Lineage-specific evidence for diversifying selection was performed using free-ratio analysis in PAML. In this instance, although some branches did exhibit dN/dS >1, the free-ratio model was not a better fit to the data than a single dN/dS ratio for the entire IL4 phylogeny.

### BALB/c and CAST/Ei Il4 cDNA

BALB/c and CAST/Ei IL4 cDNAs were prepared from Th2 cells from spleen and lymph nodes of BALB/c and CAST/Ei mice as described [54]. Total RNA was recovered using RNA STAT-60 (Tel-Test, Friendswood, TX) on day 6 of culture, following 4 hour restimulation with PMA (5 ng/ml) and ionomycin (250 ng/ml). Random hexamer-primed cDNA was generated using the Superscript II RNase H-reverse Transcriptase kit (Invitrogen). PCR was performed on resulting cDNA in 50 ul with 0.3 uM of primer (forward primer: 5'-cacagagctagtgatgggtct-3'; reverse primer: 5'-ggtggctcagtactacgagtaa-3'), 320 uM dNTP, 1.25 U AccuPrime Pfx DNA polymerase (Invitrogen) and 1× reaction buffer in a PTC-225 thermocycler (MJ Research) using the following profile: 35 cycles of 2 min 94°C, 15 sec 94°C, 30 sec 60°C, 2 min 68°C, followed by 5 min 68°C. 3' terminal adenosines were added to amplicons by incubating at 72°C for 10 min in the presence of Taq DNA polymerase (1 U) and 2 nmol dNTP. Amplicons were cloned into pCRII-TOPO cloning vector (Invitrogen) and sequenced.

### IL4 structural modelling

Polymorphic and positively selected residues were mapped onto the crystal structures of human free IL4 (1HIK), type I (3BPL) and type II IL4 receptor complexes (3BPN) using PyMol (DeLano, W.L. The PyMOL Molecular Graphics System (2002) on World Wide Web http://www.pymol.org).

### Surface plasmon resonance analysis of IL4/IL4Rα binding

Binding affinity of recombinant BALB/c and CAST/Ei IL4 (Additional Files 5, 6, 7 for purification details) to C57BL/6 IL4Rα (R&D Systems Inc) was determined by surface plasmon resonance detection using a BIAcore 2000 system (BIAcore AB, Uppsala, Sweden). Approximately 1400 RUs of IL4Rα was immobilized on a CM5 biosensor chip using amine linkage based on the manufacture's recommendation (GE Biosciences). Degassed HEPES balanced salt running buffer [HBS: 10 mM HEPES pH7.4, 150 mM NaCl, 3 mM EDTA, and 0.005% P-20 surfactant, (HBS-EP)] was used for immobilization, analyte dilutions and binding assays. The system was set to run at 25°C at a flow rate of 3 μl/min. To accommodate for the solvent bulk shift associated with IL4 dilution (coming from PBS) into HBS-EP, PBS and HBS-EP were mixed in the same dilutions and run on the machine. Graphs generated from the "blank" run were subtracted from graphs generated from IL4-containing runs. These adjustments and kinetic analyses were performed using the BIAevaluation Software ver. 3. Rate constants were calculated by applying the graphs to a 1:1 Langmuir binding algorithm. A two-minute pulse with 10 mM glycine pH 2.75 was used to regenerate the ligand/analyte.

### Cell purification

B cells were T cell-depleted splenocytes prepared by complement-mediated lysis using anti-Thy1 (J1j; American Type Culture Collection) and a combination of rabbit and guinea pig complement. As determined by FACS analysis, B cell purity from BALB/c and CAST/Ei spleens was typically 85% and 66%, respectively. CD4 T cell-enriched splenocytes were prepared by complement-mediated lysis using anti-CD8 (3.155; American Type Culture Collection), anti-HSA (J11d; American Type Culture Collection), anti-MHC class II (BP107; American Type Culture Collection) and a combination of rabbit and guinea pig complement. As determined by FACS analysis, T cell purity from BALB/c and CAST/Ei spleens was typically >91%.

### B cell CD23 upregulation assay

B cells were distributed to 24 well plates containing graded concentrations of recombinant BALB/c or CAST/Ei IL4 and incubated at 37°C for 16 hours prior to staining with PE-conjugated anti-mouse CD23 Ab (B3B4, eBioscience) and flow cytometric analysis.

### B and T cell proliferation assay

Serially diluted recombinant BALB/c or CAST/Ei IL4 was added to 96 well plates containing either 1 × 10^5 ^B cell-enriched splenocytes and 50 ng/ml of LPS (B cells) or 1 × 10^5 ^CD4 T cell-enriched splenocytes (T cells) in 200 ul RPMI 1640 medium supplemented with 10% FCS, 50 μM β-mercaptoethanol, 2 mM L-glutamine, and 100 U/ml penicillin and streptomycin. AlamarBlue (20 ul, BioSource International) was added at 24 hr and at 48 hr the extent of its reduction was determined by comparing absorption of control and test culture media at 570 nm and 600 nm. AlamarBlue reduction (AR570) was calculated as: AR570 = A570 - A600 where A570 and A600 are sample absorbances minus a media blank.

## List of abbreviations

IL2: interleukin-2; IL4: interleukin-4; IL5: interleukin-5; IL7: interleukin-7; IL13: interleukin-13; IL15: interleukin-15; IL21: interleukin-21; IL4Rα: interleukin-4 receptor alpha; IL13Rα1: interleukin-13 receptor alpha 1; Th2: T helper 2 cells; MHC; major histocompatibility complex; dN: nonsynonymous substitutions per site; dS: synonymous substitutions per site; TGFβ: Transforming growth factor beta; γc: common gamma chain; GMCSF: granulocyte macrophage colony stimulating factor; PKR: Protein kinase R; eIF2α: Eukaryotic translation initiation factor 2 alpha.

## Authors' contributions

MK performed all the immunological analyses and participated in the collection and analysis of the biophysical data. JAK performed sequence alignment and evolutionary analysis. LC isolated, cloned, sequenced and assembled rodent IL4 genomic DNA. YZ produced, purified and characterized recombinant IL4. SB carried out the collection and analysis of the biophysical data. TM created and analyzed the structural models. HSM participated in the study design, carried out the evolutionary analysis and co-wrote the manuscript. MB conceived of the study, and participated in its design and coordination and co-wrote the manuscript. All authors read and approved the final manuscript.

## Supplementary Material

Additional file 1 **IL4 polymorphisms between BALB/c and CAST/Ei**. Click here for file

Additional file 2 **Kinetic profiles of surface plasmon resonance depicting the binding of IL4 and IL4Rα**. Click here for file

Additional file 3 **IL4 alignment from 28 eutherian mammals in .phy format**. Click here for file

Additional file 4 **IL4 alignment from 16 primates in .phy format**. Click here for file

Additional file 5 **Stages of purification of recombinant IL4 purification from insect cell supernatant as measured by functional bioassay**. Click here for file

Additional file 6 **IL4 yield and fold purification**. Click here for file

Additional file 7 **Monoclonal anti-IL4 antibody BVD6 binds equivalently to recombinant BALB and CAST IL4**. Click here for file
