# Supplementary material for: Diversifying selection and functional analysis of interleukin-4 suggests antagonism-driven evolution at receptor-binding interfaces
Source: BMC Evol Biol. 2010 Jul 22;10:223. doi: 10.1186/1471-2148-10-223 (PMC3017759; doi:10.1186/1471-2148-10-223)
Supplement: Additional file 1 — IL4 polymorphisms between BALB/c and CAST/Ei. [file 1471-2148-10-223-S1.PDF]

Supporting Table 1. IL4 polymorphisms between BALB/c and CAST/Ei

	amino acid number	26	27	31		86	105	106	122
	nucleotide number	163	166	168	169	345	402	403	453
BALB/c	amino acid residue	D	K	R		P	F	R	T
	nucleotide base	G	A	A	G	A	T	C	A
CAST/Ei	amino acid residue	N	D	K		P	L	G	T
	nucleotide base	A	G	C	A	G	G	G	G

Synonymous and non-synonymous amino acid substitutions are shown in black and red, respectively. Base pair numbering is according to GenBank accession number NM_021283. Amino acid numbering is according to NP_067258.
