# Supplementary figures and images for: Diversifying selection and functional analysis of interleukin-4 suggests antagonism-driven evolution at receptor-binding interfaces
Source: BMC Evol Biol. 2010 Jul 22;10:223. doi: 10.1186/1471-2148-10-223 (PMC3017759; doi:10.1186/1471-2148-10-223)

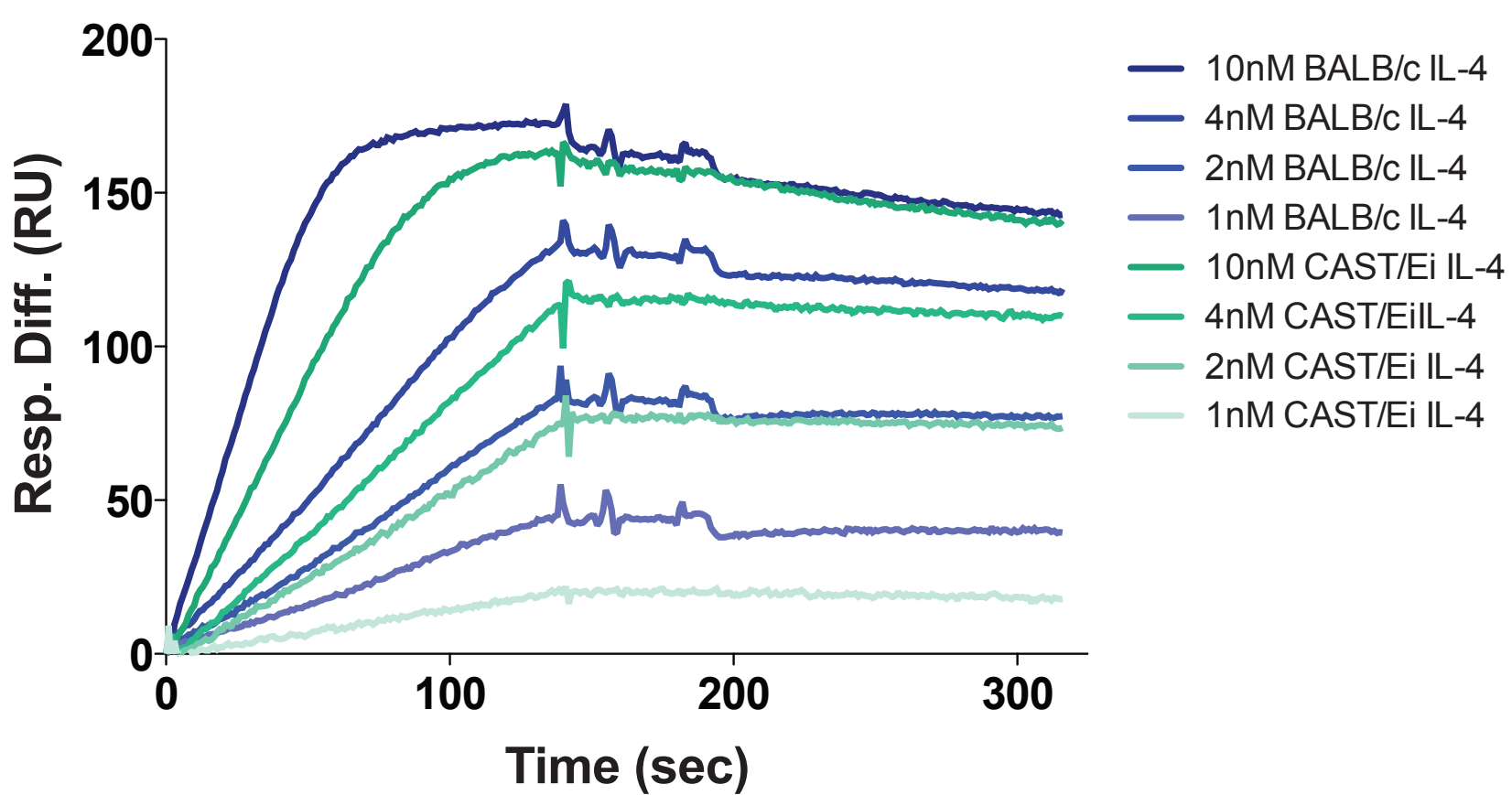

Supplement: Additional file 2 — Kinetic profiles of surface plasmon resonance depicting the binding of IL4 and IL4Rα. [file 1471-2148-10-223-S2.PDF]

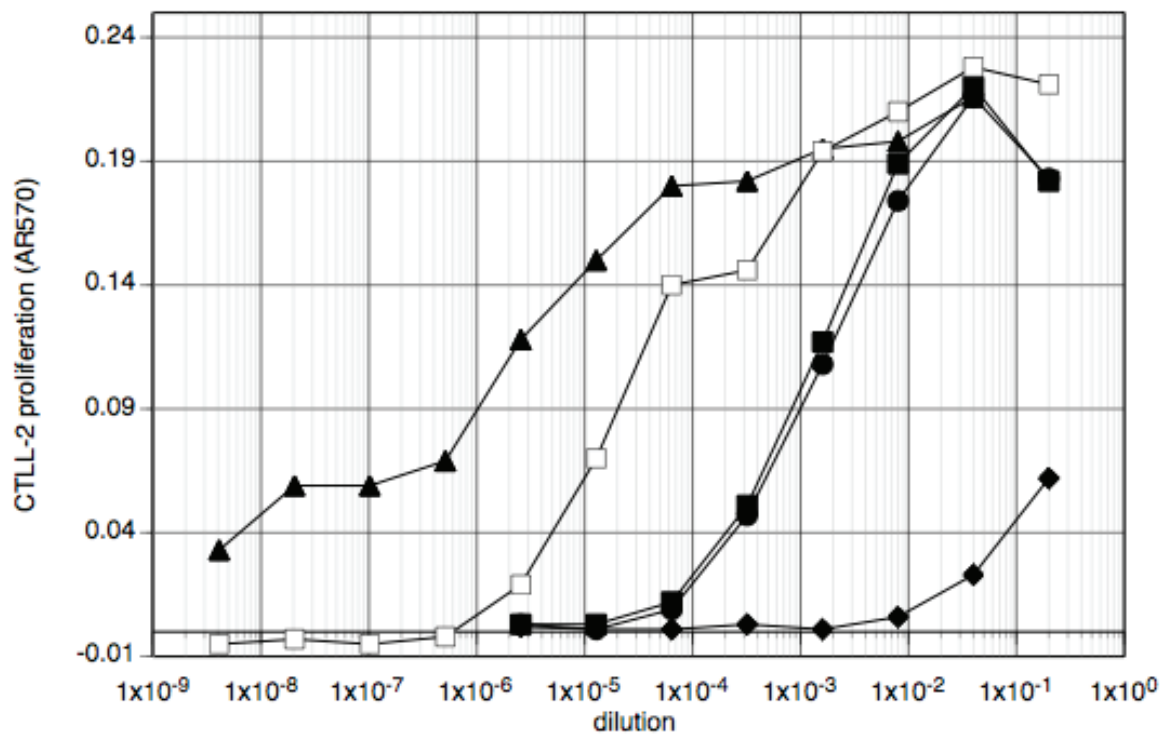

Supplement: Additional file 5 — Stages of purification of recombinant IL4 purification from insect cell supernatant as measured by functional bioassay. [file 1471-2148-10-223-S5.PDF]

Rate

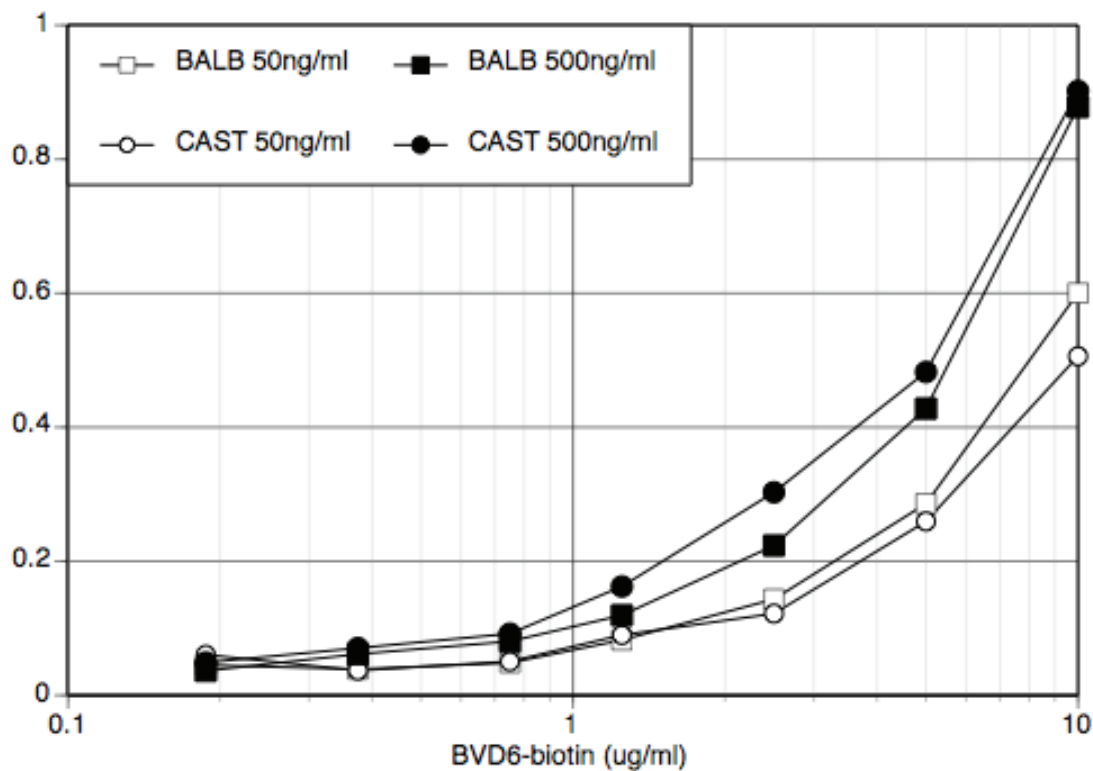

Supplement: Additional file 7 — Monoclonal anti-IL4 antibody BVD6 binds equivalently to recombinant BALB and CAST IL4. [file 1471-2148-10-223-S7.PDF]
