# Supplementary material for: Diversifying selection and functional analysis of interleukin-4 suggests antagonism-driven evolution at receptor-binding interfaces
Source: BMC Evol Biol. 2010 Jul 22;10:223. doi: 10.1186/1471-2148-10-223 (PMC3017759; doi:10.1186/1471-2148-10-223)
Supplement: Additional file 6 — IL4 yield and fold purification. [file 1471-2148-10-223-S6.PDF]

Supporting Table 2: IL4 yield and fold purification

	Volume (ml)	Protein (mg)	Total activity (Unit)	Specific activity (U/mg)	Purification (fold)	Yield (%)
Crude supernatant	2000	133.4	3.4×10^{8}	1.8×10^{3}	1	100
Purified IL4	5	0.176	2.4×10^{8}	1.4×10^{9}	7.8×10^{5}	70.6
